# Collecting duct carcinoma of the kidney: an immunohistochemical study of 11 cases

**DOI:** 10.1186/1471-2490-4-11

**Published:** 2004-09-09

**Authors:** Andrea Vecchione, Tommaso Prayer Galetti, Marina Gardiman, Hideshi Ishii, Enrico Giarnieri, Francesco Pagano, Leonard G Gomella, Carlo M Croce, Raffaele Baffa

**Affiliations:** 1 Department of Urology, Kimmel Cancer Center, Thomas Jefferson University, 1025 Walnut Street, Philadelphia, PA, 19107, USA; 2 University of Rome "La Sapienza", Ospedale Santo Andrea, Rome, Italy; 3 Department of Urology, University of Padova, Via Giustiniani 25, Padova, Italy; 4 Department of Pathology, University of Padova, Via Gabelli 3, Padova, Italy; 5 Department of Microbiology/Immunology, Kimmel Cancer Center, Thomas Jefferson University, 220 10^th ^South Street, Philadelphia, PA, 19107, USA; 6 Jichi Medical School, Center for Molecular Medicine, Division of Stem Cell Regulation/Molecular Hematopoiesis, 3311-1 Yakushiji, Minami-Kawachi, Tochigi, 329-0498, Japan

## Abstract

**Background:**

Collecting duct carcinoma (CDC) is a rare but very aggressive variant of kidney carcinoma that arises from the epithelium of Bellini's ducts, in the distal portion of the nephron. In order to gain an insight into the biology of this tumor we evaluated the expression of five genes involved in the development of renal cancer (*FEZ1/LZTS1*, *FHIT*, *TP53*, *P27*^*kip*1^, and *BCL2*).

**Methods:**

We studied eleven patients who underwent radical nephrectomy for primary CDC. All patients had an adequate clinical follow-up and none of them received any systemic therapy before surgery. The expression of the five markers for tumor initiation and/or progression were assessed by immunohistochemistry and correlated to the clinicopathological parameters, and survival by univariate analysis.

**Results:**

Results showed that Fez1 protein expression was undetectable or substantially reduced in 7 of the 11 (64%) cases. Fhit protein was absent in three cases (27%). The overexpression of p53 protein was predominantly nuclear and detected in 4 of 11 cases (36%). Immunostaining for p27 was absent in 5 of 11 cases (45.5%). Five of the six remaining cases (90%) showed exclusively cytoplasmic protein expression, where, in the last case, p27 protein was detected in both nucleus and cytoplasm. Bcl2 expression with 100% of the tumor cells positive was observed in 4 of 11 (36%) cases. Statistical analysis showed a statistical trend (P = 0.06) between loss and reduction of Fez1 and presence of lymph node metastases.

**Conclusions:**

These findings suggest that Fez1 may represent not only a molecular diagnostic marker but also a prognostic marker in CDC.

## Background

Renal cancer accounts for 3% of adult malignancies. An estimated 35,710 new cases are expected to occur in the U.S. in the current year and 12,480 patients will die of this disease [1]. The majority of renal cancers arises from the proximal tubular epithelium, with a characteristic clear or granular cell appearance by light microscopy, and is referred to as renal cell carcinoma (RCC).

Recent evidence suggests that solid renal parenchymal tumors arising in the distal portion of the nephron, such as oncocytomas, chromophobe renal cell carcinomas and collecting duct carcinomas represent an heterogeneous group of neoplasm from both clinical and biological perspectives [2]. Collecting duct carcinoma (CDC), also known as Bellini duct carcinoma, is a rare but highly aggressive renal neoplasm arising from the distal portion of the nephron, and represents approximately 2% of all the RCCs [3,4]. Clinically, CDC appears as a renal mass often accompanied by flank pain and hematuria and is frequently mistaken for RCC or transitional cell carcinoma of the renal pelvis [3,5,6]. CDC, however, can be identified based on gross, microscopic, histochemical, and immunohistochemical features. Macroscopically, CDCs are often located at the confluence of the medulla and renal pelvis, and show a characteristic gray-white-tan color, with absence of foci of necrosis and hemorrhage [7]. Histologically, CDC presents a tubulo-papillary morphology, often accompanied by desmoplasia, atypia in collecting ducts, and intratubular spread [7], features rarely seen in RCC.

Histochemically, CDC cells contain intracytoplasmic mucicarminophilic material, where RCCs do not [7]. CDCs are also positive by immunohistochemical staining with high-molecular-weight keratin and lectin, proteins typically expressed in the epithelium of the distal tubules [8,9]. Conversely, almost all other renal carcinomas express antigens widely expressed in the cells of the proximal tubules, such as low molecular weight cytokeratins and vimentin [9]. Although little is known about the genetic profile of CDCs, a DNA flow cytometry study has demonstrated aneuploidy in 90% of these tumors [5], and cytogenetics has shown frequent monosomy of chromosomes 1, 6, 8, 14, 15, and 22 [10,11].

Loss of heterozygosity (LOH) analysis in six CDCs revealed allelic loss at chromosomes 8p and 13q in 50% of the tumors, and rarely at the short arm of chromosome 3 [12,13]. This data suggests that distinct genetic alterations from those observed in RCC, in which 3p LOH is common [14], occur in the development of this rare renal tumor.

To improve our understanding of the biology of CDC and to explore the possibility that different genes may be involved in the etiology and prognosis of this neoplasm, we analyzed by immunohistochemistry eleven cases of CDC for the expression of five genes (Fez1, Fhit, p53, p27, and bcl2) often involved in the development of many common cancers. These findings were correlated with conventional clinical-pathological features including clinical outcome.

## Methods

### Patients

Eleven patients with primary CDC (two women and nine men; age range 40 to 84 years, mean 62) underwent radical nephrectomy between 1983 and 2000 in the Department of Urology, University of Padua, Italy. Tissue specimens from these tumors were registered in the Department of Pathology at the same institution. All eleven patients had an adequate clinical follow up and were included in our study. Eight of the 11 patients had metastatic disease, five with lymph node metastasis and five with distant metastases at the time of surgery. None of these eleven patients received any systemic therapy before surgery. Samples were fixed in 10% buffered formalin for routine histological processing and were stained with hematoxylin and eosin.

### Pathological study

Immunohistochemical reactions using anticytokeratin (Keratins 5, 8, 10, an 18) monoclonal antibodies, and *Ulex Europaeus Lectin*, polyclonal antibody were performed as previously described [15]. The tumors were classified histologically according to standardized criteria [3,5-7] and staged according to the guidelines of the tumor-node-metastasis (TNM) classification of malignant tumor [16].

### Immunohistochemistry

Paraffin sections containing non-neoplastic kidney as well as neoplastic areas were deparaffinized according to standard procedures followed by rehydration through graded ethanol series, and mounted on positively charged slides. Immunostaining was performed as previously described [17]. Briefly for Fez1, Fhit, p53, and p27 immunostaining, slides were immersed in citrate buffer [0.01 M sodium citrate (pH6.0)] and heated in a microwave oven at 600 W (three times for 5 min each) to enhance antigen retrieval. For retrieval of Bcl2 oncoprotein, we used the target retrieval solution, high pH from DAKO (Carpinteria, CA, USA) per manufacturers instructions. The primary antibodies used in this study were: anti-Fez1 rabbit polyclonal antibody [17], anti-Fhit rabbit polyclonal antibody (Zymed Laboratories, San Francisco, CA) at 1:1,000 dilution, anti-p53, anti-p27, anti-Bcl2 (DAKO, Carpinteria, CA, USA) at dilution of 1:25, 1:50, and 1:40 respectively, as specified by the manufacturers. The primary antibodies were omitted and replaced with pre-immune serum in the negative controls. Sections were reacted with biotinylated anti-rabbit or anti mouse antibodies and streptavidin-biotin-peroxidase (Histostain-SP Kit; Zymed laboratories, San Francisco, CA). Diaminobenzidine (DAB) was used as a chromogen substrate to visualize staining. Finally, sections were washed in distilled water and weakly counterstained with Harry's modified hematoxylin. The immunostaining was evaluated by two pathologists in a blinded fashion (A.V.; R.B.). For statistical analysis, cases were scored as positive if they had more than 20% (p53) or more than 40% (Fez1 and p27) of positive cells. Fhit and bcl2 cases showed either all positive or all negative cells and were scored accordingly.

### Statistical analysis

We evaluated the association between each marker's expression (positive or negative) and each clinical-pathological outcome (metastasis and stage) with Fisher's exact test. For survival, we used the Kaplan-Meier method and log-rank test, as well as Cox proportional hazards regression.

## Results

### Pathological study

All cases showed several common microscopic features. The main tumor consisted of a tubulo-papillary carcinoma showing pleomorphism and a high mitotic rate with several bizarre mitotic figures, and presence of small cystic spaces surrounded by a highly desmoplastic stroma.

Two cases showed a predominant papillary pattern accompanied with dilated and atypical changes in the epithelium of the collecting ducts in the adjacent renal medulla, which was the most convincing feature supporting a collecting duct origin of these tumors. Metastases, when available for examination, were histologically similar to the infiltrating part of the primary tumors. Immunocytochemically, tumor cells from all cases examined did not express keratin 10, while strong positivity to anti-keratin 5, 8, and 18 antibodies, and anti-UEL antibody was observed. The positivity to high molecular weight keratin and UEL further substantiate the diagnosis of CDC. The clinical-pathological features and protein expression of the study's 11 cases are shown in Table 1.

### Fez1 expression

**Figure 1:**
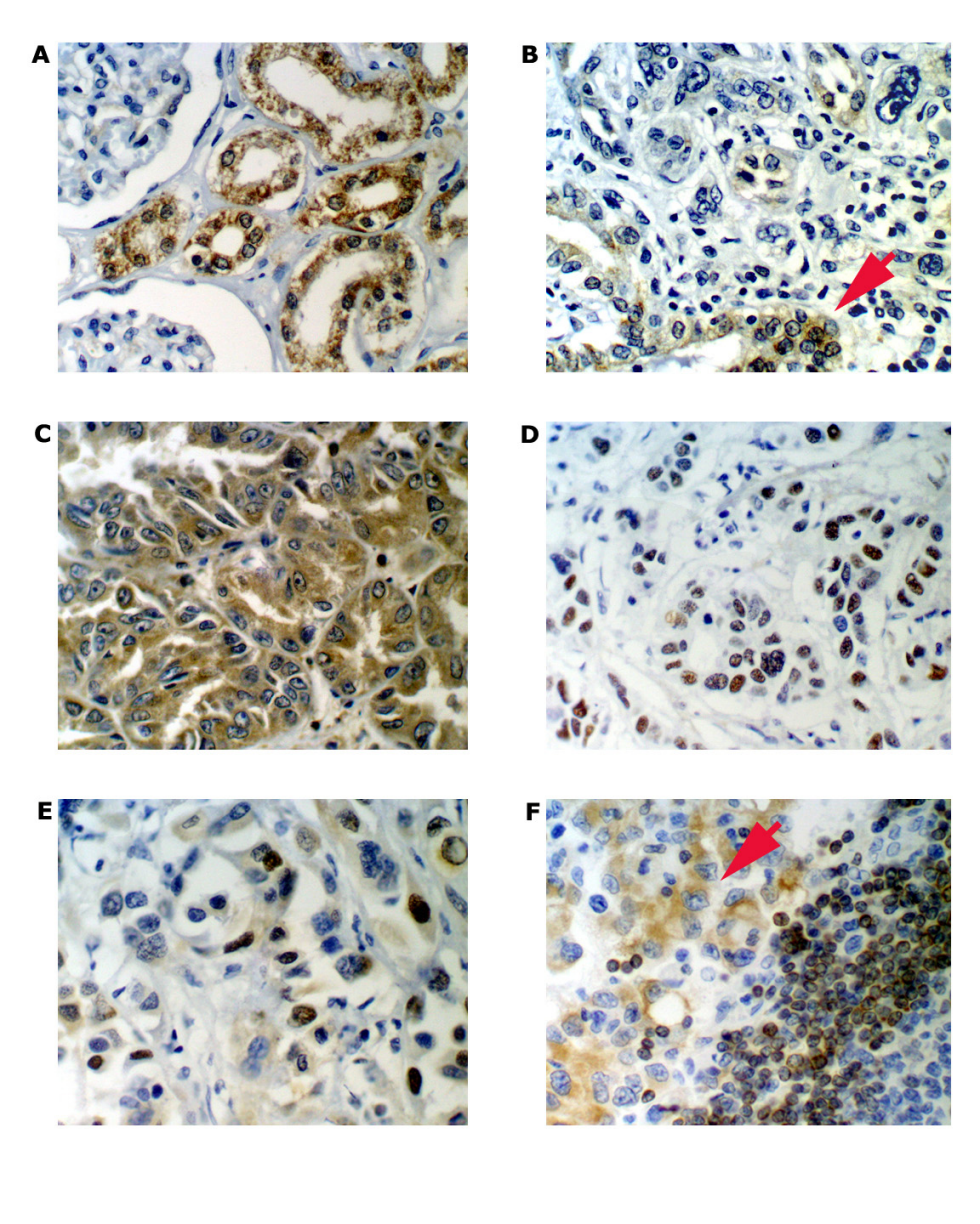
Representative examples of immunohistochemical analysis of Fez1, Fhit, p53, p27 and bcl2 proteins in primary Bellini's duct carcinoma of the kidney. **A**, Normal renal parenchyma shows uniform, cytoplasmic positive staining for Fez1 (×400). **B**, Fez1 protein is absent in this CDC (Case 1; ×400). An adjacent dysplastic distal duct (*red arrow*) shows strong Fez1 immunoreacticity. **C**, CDC section showing diffuse cytoplasmic staining for Fhit protein (Case 2; ×400). **D**, CDC neoplastic glands display strong nuclear immunoreaction for p53 oncoprotein (Case 5; ×400). Case 9 shows p27 immunoreactivity in both nucleus and cytoplasm **(E) **and bcl2 cytoplasmic immunostaining, *red arrow ***(F) **(×400).

**Figure 2:**
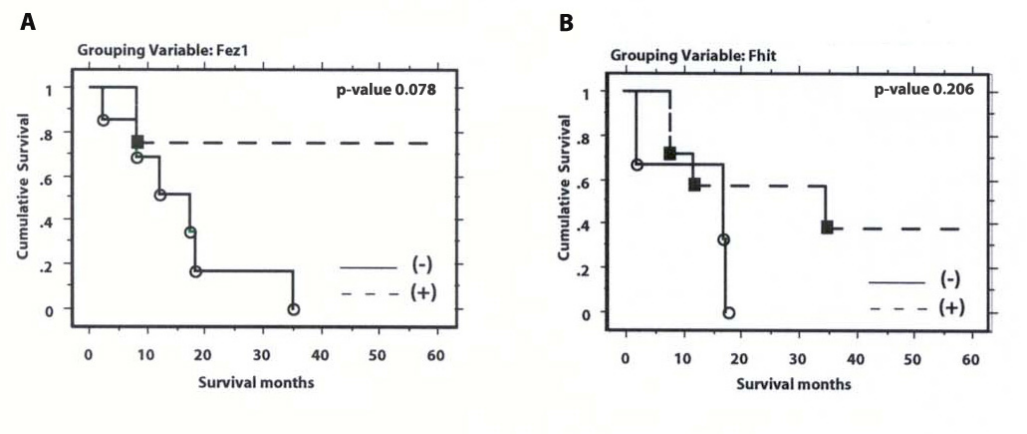
Kaplan-Meier survival curves and associated log-rank test p-values for of eleven patients with collecting duct carcinoma of the kidney based on Fez1 (A), and Fhit (B) expression. (+); positive expression; (-) = negative expression; ■ and **o **= Event times.

Our results showed that Fez1 protein expression was undetectable in six of the eleven (55%) cases. One case showed a substantial reduction of expression with 60% of the tumor cells negative, while the remaining four showed diffuse positivity for Fez1 immunostaining (Figures 1A and 1B). Overall 64% of the cases showed absence or reduction of Fez1 expression. Loss and reduction of Fez1 were correlated with a higher prevalence of lymph node metastases (71% vs. 0%, p = 0.061), although the same was not true for distant metastases or tumor stage. Fez1-negative tumors also tended to have higher mortality than Fez1-positive tumors. Median survival time was estimated to be 17 months for the former group, while median survival was not reached for the latter group during the course of the study (i.e., fewer than half of the patients died) (p = 0.078; Figure 2A). This corresponds to an estimated 5-fold increase in mortality risk for the Fez1-negative CDCs (hazard ratio of 5.5).

### Fhit expression

Fhit protein was absent in three of eleven cases (27%), and the remaining eight showed diffuse immunoreactivity (Figure 1C). Reduction of Fhit protein expression did not correlate with any of the clinicopathological features of the tumors. There was a tendency for Fhit-negative cases to have worse survival than Fhit-positive cases (median survival times of 17 vs. 35 months, respectively; hazard ratio of 2.7), although the difference was not statistically significant (p = 0.206: Figure 2B).

### p53 expression

p53 protein was found to be overexpressed mainly in the nucleus in four of eleven cases (36%) (Figure 1D). One of the four positive cases showed overexpression in 85% of the cancer cells, whereas the remaining three showed 20%, 30% and 50% of positive cancer cells, respectively. p53 was not detectable in seven of eleven cases (63%) as well as in the normal renal epithelium adjacent to tumor. Overexpression of p53 was not related to histologic grade, tumor stage, lymph node status, and survival.

### p27 expression

Immunoreactivity for p27 was observed in the nuclei of most glomerular and tubular cells in the normal kidney. p27 immunostaining in CDC showed high variability. Protein expression was absent in five of eleven cases (45.5%). All the remaining six carcinomas showed a high percentage (> 40%) of p27 positive cells. The expression of p27 protein was detected exclusively in the cytoplasm in five of the six positive cases (90%) while a mixture of nuclear and cytoplasmic protein staining was observed in the last case (Figure 1E). Overall, we observed lack or subcellular compartmentalization of p27 in 90% of the cases. The status of p27 did not correlate to any of the clinical-pathological parameters tested.

### Bcl2 expression

Bcl2 protein expression in 100% of the CDC tumor cells was observed in four of eleven cases (36%) (Figure 1F), while the remaining seven (64%) were negative. Statistical analysis did not reveal any significant correlation between Bcl2 expression and other clinical-pathological parameters.

Clinical-pathological features as well as immunohistochemistry results are listed in Table 1. The proportion of marker expression ranged from 36% (Fez1 and Bcl2) to 73% (Fhit). Marker expression tended to be positively correlated, with the correlation between Fhit and p27 being the strongest (0.67), followed by that between Fhit and Fez1 and Fhit and Bcl2 (both 0.46).

## Discussion

The application of the most recent molecular cytogenetic techniques revealed that renal parenchymal tumors can be classified into distinct subtypes based on the combination of specific genetic alterations [18]. The pathological and immunohistochemical description as well as the cytogenetic abnormalities support the hypothesis that CDC is more similar to urothelial carcinoma than to clear cell carcinoma of the kidney. Indeed, whereas allelic deletion of the short arm of chromosome 3 is considered a genetic hallmark of clear cell carcinoma, LOH at chromosomes 8p, 9p, and 17p has been frequently described in both transitional cell carcinoma and CDC. Here, we have reported the results of our immunohistochemical analysis of the expression of five genes (*FEZ1*, *FHIT*, *P53*, *P27*^*kip*1^, and *BCL2*) in a relatively large series of CDCs (eleven cases), considering the rarity of this tumor.

*FEZ1/LZTS1 *(leucine zipper tumor suppressor 1) is a putative tumor suppressor gene located at 8p22 [19]. Studies have indicated this chromosomal region is the location of an important tumor suppressor gene (TSG) [20]. LOH at 8p has been described in 50% of the CDCs studied [12], suggesting that a TSG in this region may also play a role in the development of this rare tumor. In our study, we found loss of Fez1 expression in the majority of CDCs studied and a correlation with the presence of lymph node metastasis. Furthermore, lack of Fez1 protein correlated with a poorer prognosis in 90% of patients with median survival of 17 months.

*FEZ1 *encodes a 67-kDa leucine-zipper protein with a region of similarity to cAMP-dependent activated protein [19]. Mutations of *FEZ1 *gene have been reported in several solid tumors, including prostate, breast, esophageal, and gastric carcinomas [17,19]. In addition, reduced Fez1 expression is associated with high-grade transitional cell carcinoma of the bladder [21]. Recent studies have shown that the introduction of *FEZ1 *into Fez1 null cancer cells reduced cell growth with the accumulation of cells at late S to G_2_/M phase of the cell cycle. Conversely, inhibition of Fez1 expression stimulated cells growth [22]. Furthermore, LOH at the chromosomal region where the *FEZ1 *gene lies (8p21-22) has been also associated with the invasive behavior of breast cancer [23] and with prostate cancer progression [24]. These data are consistent with an important role of *FEZ1 *in several human cancers including CDC.

The tumor suppressor gene *FHIT *maps to the short arm of chromosome 3 (3p14.2), encompasses the common *FRA3B *fragile region, and encodes for a protein of 16.8 kDa, with diadenosine triphosphate hydrolase activity [25]. Reduction of Fhit protein expression as consequence of alteration of the *FHIT *gene has been observed by immunohistochemistry in many types of cancers [26,27]. Although Shoemberg et al. did not detect LOH involving chromosome 3p [12], Hadaczek et al. reported LOH at 3p in two cases of CDC [13]. The same authors described a correlation between reduced Fhit expression and 3p allelic loss in renal carcinomas, particularly in CDCs [28]. While in our study Fhit inactivation does not seem to be a common event in CDC, an involvement of the *FHIT *gene in tumorigenesis of this rare tumor cannot be excluded.

The *TP53* tumor suppressor gene maps to chromosome 17p13.1 and plays a major role in DNA transcription, cell growth, proliferation and apoptosis process [29]. In normal cells, expression of wild type p53 protein is generally below the detection level when studied by immunohistochemical method. However, p53 gene point mutations occur frequently (22–76%) in different solid neoplasms. Mutated p53 protein, being more resistant to degradation, accumulates in the cells and can be detected by immunohistochemistry. Although an association between p53 protein overexpression and tumor stage, grade and survival has been observed in RCC [30] our data suggest that involvement of p53 alterations does not occur with the same frequency in CDC.

P27 is a member of the universal cyclin-dependent kinase inhibitor (CDKI) family. The expression of this important protein is regulated by cell to cell contact inhibition as well as by specific growth factors, such as transforming growth factor (TGF-β). In addition to its role as a CDKI, p27 is considered a putative tumor suppressor gene, a major regulator of drug resistance in solid tumors, and a promoter of apoptosis. p27 acts also as a safeguard against inflammatory injury and has a role in cell differentiation [31]. It has been suggested that loss of the p27 negative cell cycle regulation may contribute to oncogenesis and tumor progression in several tumor types. In renal cell carcinoma, Kamai et al. reported that low level of p27 protein was associated with tumor invasion and unfavorable prognosis, suggesting p27 as a powerful prognostic marker for survival in urinary tract cancer [32]. Masuda et al. indicated that p27 has an independent predictive prognostic value for transitional cell carcinoma of the renal pelvis [33]. Our results show that p27 loss or subcellular compartmentalization represents a frequent feature in CDCs. Previous studies have noted that cytoplasmic localization of p27 lead to an inactivation of its normal function as negative cell cycle regulator [34].

Nevertheless, we did not find statistical correlation to assess its involvement in CDC biology, possibly due to the limited number of tumors studied.

The proto-oncogene *BCL2*, implicated in the regulation of cell death by inhibiting apoptosis, seems to be vital in normal kidney morphogenesis. In fact, Bcl2 deficient mice develop polycystic kidneys characterized by dilated proximal and distal tubules [35]. High levels of Bcl2 protein expression have been found in many different types of cancer, suggesting a possible role for Bcl2 deregulation of apoptosis and malignant tissue transformation. Expression of Bcl2 has also been associated with poor prognosis in patients with various cancers including prostate cancer [36]. In the present study, Bcl2 expression was not associated with any clinical-pathological variables.

Our results suggest a potential association between FEZ1 expression and CDC pathology and prognosis. No similar patterns were seen for any of the other markers studied. Even so, the statistical power of the study was limited and negative findings should not be construed as evidence that these markers are not important. Rather, a larger study would need to be carried out to further investigate their role in CDC.

## Conclusions

Our results suggest that Fez1 expression may be associated to both clinical-pathological features and survival in patients with CDC. *FEZ1 *gene alterations may be linked to the high frequency of LOH found at 8p22, where *FEZ1 *resides. The lack of similar association for the other four genes studied may be due to the low statistical power of the study.

## Competing interests

None declared.

## Abbreviations

CDC, Collecting Duct Carcinoma; RCC, renal cell carcinoma; LOH, loss of heterozygosity

## Authors' contributions

A.V. carried out the immunohistochemical analysis and reviewed the slides, he also contributed to the draft of the manuscript. T.P.G. was responsible for the clinical study. M.G. carried out the original histopathological diagnosis. H.I. participated in the immunohistochemical analysis and statistical analysis. E.G. participated in the immunohistochemical analysis and statistical analysis. F.P. was responsible for the clinical study. L.G.G. participated in designing the study and in drafting the manuscript. C.M.C. participated in designing the study. R.B. participated in the original design and coordination of the study, and in writing the manuscript

## Pre-publication history

The pre-publication history for this paper can be accessed here:



## Figures and Tables

**Table 1:** Clinical-pathological features and protein expression.

**PZ ID**	**T**	**N**	**M**	**STAGE^a^**	**Fez1**	**Fhit**	**P27**	**Bcl2**	**P53**	**Survival (months)**
**1**	3	1	0	3	-*	-	-	-	+^1^	17^+^
**2**	4	2	0	4	-	+	-	+	-	8^+^
**3**	3	0	1	4	-	-	-	-	-	2^+^
**4**	4	2	1	4	-	+	Cyt	-	+^2^	35^+^
**5**	3	0	0	3	-	+	Cyt	-	+^3^	12^+^
**6**	3	1	1	4	-	-	-	-	-	18^+^
**7**	2	0	0	2	+	+	-	-	-	58^#^
**8**	3	0	0	3	+	+	Cyt	+	+^4^	55^#^
**9**	3	0	1	4	+	+	Cyt^	+	-	8^+^
**10**	2	0	1	4	+	+	Cyt	-	-	16^#^
**11**	3	1	0	3	-	+	Cyt	+	-	7^#^

^a ^staged following the guidelines of the tumor-node-metastasis (ref 16)

* Tumor showed 60% of the cells negative for Fez1 expression

^ Nuclear p27 was also detected in this case

^1,2,3,4^: Showed 85,20,30,50 % of the cells positive for p53 expression, respectively

^+ ^Dead

^#^Alive
